# Development and validation of the PACPN: Parents’ Attitudes Towards Crying Pediatricians and pediatric Nurses questionnaire

**DOI:** 10.1007/s00431-025-06030-x

**Published:** 2025-02-17

**Authors:** M. Foijer, B. Spek, M. A. de Vos, A. A. E. Verhagen, J. L. Aris-Meijer

**Affiliations:** 1 Department of Pediatrics, University Medical Center Groningen, Beatrix Children’s Hospital, University of Groningen, Groningen, The Netherlands; 2 Department Research Group Healthy Ageing, Allied Health Care and Nursing, Hanze University of Applied Sciences Groningen, Groningen, the Netherlands; 3 Department of Epidemiology & Data Science, Amsterdam University Medical Center, University of Amsterdam, Amsterdam, The Netherlands; 4 Department of Pediatrics, Emma Children’s Hospital, Amsterdam University Medical Center, Amsterdam, Netherlands

**Keywords:** Crying, Pediatricians, Pediatric nurses, Pediatric palliative care, Parents

## Abstract

Crying by healthcare professionals in the medical setting is a common but understudied phenomenon. We aimed to develop a questionnaire which measures the attitudes of parent towards crying pediatricians and pediatric nurses. We assessed reliability and validity in a group of parents of children who are living with, have died from or survived a life-limiting or life-threatening-condition. The development of the PACPN was based on modification of an existing questionnaire and expert input. In a cross-sectional-design, we assessed reliability and validity for both pediatricians and pediatric nurses. Dimensionality was assessed using principal component analysis (PCA). Cronbach’s alphas were calculated for each subscale. For construct validity, participants were asked to rate an additional question regarding the goal to measure parents’ attitudes towards crying pediatricians/pediatric nurses. We hypothesized that a higher score would have a strong positive correlation with the total score of the PACPN. At the end of the questionnaire, participants were asked to rate and comment the completeness. The developed 25-item questionnaire was completed by 116 parents. The PCA revealed two dimensions: (1) family’s circumstances; (2) personal circumstances of the pediatrician/pediatric nurse. Internal consistency was good (pediatricians, .81–.93; pediatric nurses, .83–.93). The hypothesis regarding construct validity was confirmed (Spearman’s rho = .71–.75). The completeness score was 7.7 (min–max 1–10, SD = 1.51).

*Conclusion*: The PACPN showed good internal consistency and some degree of construct validity. We assume that by adding some items with nuance to the situation and the degree of crying the completeness of the questionnaire will improve.

**Table:** 

**What is Known:** *• Crying by HCPs in the medical setting, such as the hospital is common but HCPs have different attitudes towards this.* • *A questionnaire on crying physicians and nurses is available for assessing HCPs attitudes.*	
**What is New:** *• The PACPN questionnaire is a simple tool for assessing parents’ attitudes towards crying pediatricians and pediatric nurses.* • *The PACPN showed good internal consistency and some degree of construct validity.*

**What is Known:**

*• Crying by HCPs in the medical setting, such as the hospital is common but HCPs have different attitudes towards this.*

• *A questionnaire on crying physicians and nurses is available for assessing HCPs attitudes.*

**What is New:**

*• The PACPN questionnaire is a simple tool for assessing parents’ attitudes towards crying pediatricians and pediatric nurses.*

• *The PACPN showed good internal consistency and some degree of construct validity.*

## Introduction

Crying by healthcare professionals (HCPs) in the medical setting is a common but understudied phenomenon [1, 2]. Prior research showed that it was associated with personal circumstances of HCPs and those of their patients, and occurred both in absence and presence of patients or their families [1]. In a large study in the Netherlands (*n* = 776), approximately half of the physicians cried in the last year at least once in the working environment [1].

HCPs differ in their opinions on the acceptability of their own crying or that of their colleagues [1–4]. Physicians and medical interns have various negative attitudes towards their crying, e.g., seeing this as inappropriate or unprofessional behavior, perceiving this as ineffective for their work process or even considering this as a sign of weakness [1, 2]. Of all studied disciplines, gynecologists, and pediatricians cry most often at their place of work, both in the absence and in the presence of patients [1]. The death of a baby or child is the main reason for crying [2, 3]. Conversations with parents about their child’s impending death can be extremely emotionally challenging for HCPs [5]. HCPs working in the pediatric palliative care experience caring for dying children as a stressful job that is accompanied with personal pain and inadequate emotional support [6]. Moreover, HCPs working in the PICU (Pediatric Intensive Care Unit) or NICU (The Neonatal Intensive Care Unit) experience higher moral distress than HCPs working in the adult ICU [7].

Parents who deal with life-threatening conditions and possibly even the imminent death of their child value HCPs who show their emotions appropriately and have a clear need for emotional support [8–12]. Additionally, parents who believe that communication suffered due to a lack of emotional support describe the attending nurses and doctors as uninterested, insensitive, or unempathetic [13]. A recent Dutch study underlines parents’ need for more empathic communication [14]. Parents experience insensitive HCPs during their child’s dying process as one of the most distressing factors [11, 15]. Parents’ perception of uncaring emotional attitudes displayed by HCPs during or after the death of their child may also have a detrimental effect on their early and long-term grief [16, 17].

According to parents, nurses seem to provide more emotional support to parents than doctors [8, 13, 15]. Compared to doctors, they also appear to have less difficulty showing their emotions [3, 4]. Although crying by nurses seems to be generally more accepted by both doctors and nurses [3], little is known on the parents’ attitudes towards the acceptability of crying pediatricians and pediatric nurses and if this differs between both HCPs. In general, not being able to cry can be associated with lower levels of empathy [18]. More specifically, prior research showed that crying with parents at the time and after the child’s death, was perceived as emotionally supportive and empathetic, which was found to be associated with higher trust from patients and beneficial effects on parental bereavement [2, 11, 16, 17]. HCPs struggle with how to express their emotions and are looking for a balance between professional distance and empathy [1]. Gaining more insight in parents’ attitudes towards crying could strengthen HCPs in how they support parents and maintain themselves while providing care. Acquiring this knowledge is an important first step for informing future research on this topic. To date, however, there is no valid questionnaire that measures these attitudes. The aim of this study is to develop a questionnaire which measures these attitudes, and to assess its reliability and validity in a representative group of parents of children who are living with, have died from or have survived a life-limiting condition (LLC) or life-threatening condition (LTC).

## Method

### Design

To assess the reliability and validity of this questionnaire, a cross-sectional design has been used. Ethical approval was obtained by the Medical Ethics Review Board for non-WMO research (CTc) UMCG (Universitair Medisch Centrum Groningen).

### Recruitment

In September and October 2022, Dutch-speaking parents of children from 0–18 years who are living with, have died from or have survived an LLC or LTC were invited through the newsletter, website, and social media channels of the Dutch Centre of Expertise in Children’s Palliative Care, the Child and Hospital Foundation and the Association of Parents of a Deceased Child. The invitation included a brief introduction to the study and the direct link to the survey. Conform the recommendations of de Vet et al. [19], a sample of minimum 50 participants was strived for.

### Measurements

#### Background variables

All data were collected with REDCap. The following background characteristics were collected: participants’ gender, age, and education level as well as their children’s gender, age and main diagnosis, and the time since diagnosis or death. In addition, information on the participants experiences with crying pediatricians and/or pediatric nurses and the potential reasons for crying by pediatricians and pediatric nurses according to parents were collected.

#### Original questionnaire

The original questionnaire was developed for physicians and medical interns. It measured their occurrence of crying in the working environment and their attitudes towards it [1]. This questionnaire consisted of 2 parts. Part 1 consisted of several background characteristics of the HCPs and nine propositions concerning attitudes towards crying during patient contact. Part 2 consisted of 63 propositions, 18 open/closed questions, and went into more detail on the topic [1].

#### Development of the PACPN questionnaire

The developers gave their permission for the modification of the original questionnaire which consisted of three steps: (1) deleting and/or rephrasing the questions in such a way that all items were appropriate for participating parents to rate their attitudes towards crying by pediatricians and pediatric nurses. (2) To obtain face and content validity, the first draft of the modified questionnaire was presented to an expert panel consisting of two parents, a clinical psychologist working in pediatric palliative care and a clinical epidemiologist. The written feedback was used to improve the questionnaire (e.g., adding/deleting questions; correcting, or deleting mistakes, vague, or ambiguous questions; and recommendations regarding the length). (3) The expert panel tested the questionnaire on the workability of the online questionnaire and time necessary to complete. There were no comments about the workability of the questionnaire, and it took them about 5–10 min to complete.

#### PACPN questionnaire

This resulted in a 29-item questionnaire. A Likert scale from 1 (strongly disagree) to 7 (strongly agree) was used. Responses to negative questions were coded reversely to avoid bias in the total score. A total score can be composed by adding all sub-scores together.

**Fig. 1:**
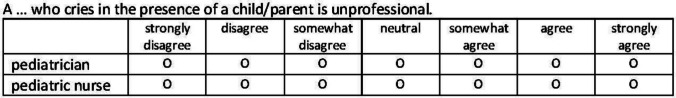
Example of one of the questions in the PACPN

For each question, the participants were asked to what extent they agreed with pediatricians and pediatric nurses (see Fig. 1 for an example of one of the questions).

In the questionnaire crying was defined as “The broad range of emotional expressions from just moist eyes to sobbing and crying out loud and could be a reaction of, for example, sadness or joy” [20].

#### Construct validity

To measure construct validity, all respondents were asked an additional question after completing the questionnaire: to what extent (0–10) do you have a positive attitude towards crying pediatricians or pediatric nurses? We hypothesized that a higher score on this question would have a strong positive correlation (*R* = 0.7–1.0) with the total score of the PACPN questionnaire.

#### Completeness of the PACPN

To gain insight in the perception of completeness, participants were asked if they felt that the questionnaire measured their thoughts about crying by pediatricians and pediatric nurses on a scale of 1–10. Finally, respondents could add additional commentary regarding the questionnaire.

### Statistical analyses

Frequencies, percentages, medians, and interquartile ranges were calculated to describe parents’ and children’s characteristics. To establish whether or not the questionnaire was unidimensional, we used a principal component analysis (PCA) with oblique rotation. Before interpreting the rotated factor loadings, Kaiser-Meyer-Olin Measure of Sampling Adequacy (KMO) and Bartlett’s Test of Sphericity were checked on adequacy of the sample for PCA. In accordance with the recommendations of Kaiser, only items with KMO > 0.50 were accepted [21]. A parallel analysis and scree plot examination were used to indicate how many factors should be retained. Factor loadings > 0.40 were considered acceptable. We assessed the internal consistency of each dimension with Cronbach’s alpha coefficient. In general, a well-accepted guideline for the value of Cronbach’s alpha is between 0.70 and 0.90 [19]. All analyses on the internal consistency and validity of the questionnaire were conducted separately for the questions on pediatricians and on pediatric nurses. All data were analyzed using R (version 3.5.1) with RStudio (version 1.1.463) using the data.table, psych, and GPArotation packages.

## Results

### Sample characteristics

**Fig. 2:**
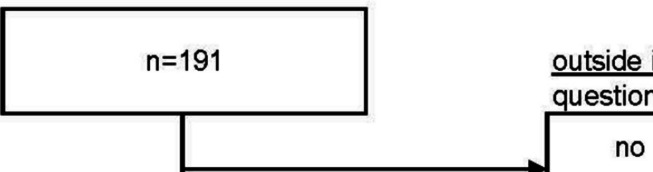
Flowchart of respondents

Of the 191 participating parents who started the questionnaire, 33 were excluded based on the selection criteria (Fig. 2). One hundred sixteen out of these 158 parents (73%) completed the questionnaire.

The characteristics of these parents and their children are shown in Table 1. Of all parents, 92% were mothers (*n* = 105). The main diagnosis of the children was cancer (*n* = 31, 27%). Fifty-seven (49%) children had deceased. Sixty-seven parents (58%) reported that they themselves had experiences with crying pediatricians and/or pediatric nurses (Table 1).

**Table 1:** Characteristics of parents and their children

Characteristics of parents (*n* = 116)*	
Gender	
Male	9 (8)
Female	105 (92)
Age (median, IQR)	43 (38–47)
Education	
Low^a^	2 (2)
Middle^b^	28 (24)
High^c^	86 (74)
Medical or nursing trained	
Yes	30 (26)
No	85 (74)
Experience with crying HCPs	
Experience with a crying pediatrician	13 (11)
Experience with a crying pediatric nurse	21 (18)
Experience with both HCPs	33 (28)
No experience	49 (42)
Characteristics of the children (*n* = 116)*	
Gender	
Male	57 (53)
Female	51 (47)
Age at diagnosis (median, IQR)	1 (0–4)
Diagnosis	
Cardiac	11 (10)
Congenitally acquired	13 (12)
Chromosomal/syndromic	5 (4)
Metabolic	12 (11)
Neurologic/neuromuscular	10 (9)
Oncologic	31 (27)
Premature	5 (4)
Pulmonal	2 (2)
Other	17 (15)
Undiagnosed	7 (6)
Deceased	
Yes	57 (49)
No	59 (51)

*Data are given as number (percentage) of respondents unless otherwise indicated. Percentages may not total 100 because of rounding. ^a^low: primary school, lower secondary general education, lower vocational education; ^b^middle: higher secondary general education, intermediate vocational education; ^c^high: higher vocational education, university

### Reliability and validity of the PACPN questionnaire

#### Pediatricians

Based on KMO < 0.5 or factor loading < 0.4, four items were deleted (Table 2). The total KMO of the final 25-item questionnaire was 0.89, all 25 items were > 0.70. Bartlett’s test of sphericity was significant (*χ*^2^ = 605.137, *P* < 0.001). The parallel analysis and scree plot indicated a two-dimension solution. Table 2 shows the factor loadings after oblique rotation. Twenty-one items cluster on the first dimension representing the “family circumstances.” The second dimension consists of four items representing the “personal circumstances of the pediatrician.” Together, they explained 50% of the variance. The fit of the model was 0.96. Cronbach’s alpha showed an internal consistency of 0.93 for the first and 0.81 for the second dimension.

**Table 2:** Summary of principal component analysis for the pediatricians (*N* = 113)

	Oblique rotated factor loadings	
Item	Child’s/parents’ circumstances	Pediatrician’s circumstances
The emotions of a pediatrician in the relationship with children/parents should receive more attention in education.*		
Without the presence of the child/parent, I think it’s appropriate for the pediatrician to cry because of the child/parent’s bad situation.*		
I believe that the tears of a pediatrician during a child/parent contact are experienced as very stressful by the pediatrician.**		
Crying by a pediatrician in the presence of a child/parent might increase the risk of burn out.**		
A pediatrician may also cry during a child/parent contact	** − 0.84**	
It is disturbing when a pediatrician cries during a child/parent contact	**0.82**	
Tears of a pediatrician may be visible to the child/parent	** − 0.80**	
In contact with a child/parent, a pediatrician should be as authentic as possible and be him/herself, even if this means that (s)/he sometimes has to cry	** − 0.77**	
A pediatrician who cries in the presence of a child/parent is unprofessional	**0.76**	
A pediatrician should feel free to cry during a child/parent contact	** − 0.75**	
A pediatrician who cries during child/parent contact is not suitable as pediatrician	**0.74**	
I think it’s appropriate for a pediatrician to cry in the presence of a child/parent because of the bad situation of the child/parent	** − 0.72**	
When a pediatrician cry (s)/he cannot control his/her emotions	**0.72**	
I feel uncomfortable when a pediatrician cries during a child/parent contact	**0.69**	
During a child/parent contact, professional distance by a pediatrician is more important than empathy	**0.68**	
A pediatrician who cries in the presence of a child/parent can be a good empathic reaction	** − 0.66**	
I think it’s appropriate for a pediatrician to cry in the presence of a child/parent	** − 0.66**	
When a pediatrician cries in the workplace, (s)he cannot handle the job	**0.58**	
When a pediatrician cries during a child/parent contact, (s)he shows inappropriate involvement	**0.58**	
A pediatrician who cries in the presence of a child/parent has greater risk to make mistakes and/or wrong decisions	**0.57**	
A good pediatrician should always take distance from the child/parent and his/her problems	**0.53**	
A pediatrician who cries in the presence of a child/parent makes him/herself ridiculous	**0.52**	
Tears of a pediatrician in the presence of a child/parent could be important for the contact between child/parent and pediatrician	** − 0.50**	
When a pediatrician starts crying, I try to get him/her to stop immediately	**0.50**	
I ignore the crying of a pediatrician during a child/parent contact	**0.41**	
I think it’s appropriate for a pediatrician to cry in the presence of a child/parent because of negative performance feedback		**0.87**
I think it’s appropriate for a pediatrician to cry in the presence of a child/parent because of a conflict in the work setting		**0.83**
I think it’s appropriate for a pediatrician to cry in the presence of a child/parent because of work overload		**0.78**
I think it’s appropriate for a pediatrician to cry in the presence of a child/parent because of private circumstances		**0.70**
Eigenvalues	9.38	2.91
% of variance	38.00	12.00
α	0.93	0.81

*Items deleted based on KMO < 0.5. **Items deleted based on factor loading < 0.4

#### Pediatric nurses

The same four questions were deleted in the questions regarding pediatric nurses as for the pediatricians, because KMO was < 0.5 or factor loading was < 0.4 (Table 3). The final 25-questionnaire showed a total KMO of 0.86 and all items were > 0.63. Bartlett’s test was significant (*χ*^2^ = 1537.535, *P* < 0.001). The factor loadings after oblique rotation are shown in Table 3. In accordance with the questions on pediatricians, the fit of the model was 0.96. Both dimensions consisted of the same items and represented the same categories, i.e., the family’s circumstances (Cronbach’s alpha 0.93) and the personal circumstances of the pediatric nurse (Cronbach’s alpha 0.83). Together they explained 48% of the variance.

**Table 3:** Summary of principal component analysis for the pediatric nurses (*N* = 109)

	Oblique rotated factor loadings	
Item	Child’s/parents’ circumstances	Pediatric nurse’s circumstances
The emotions of a pediatric nurse in the relationship with children/parents should receive more attention in education.*		
Without the presence of the child/parent, I think it’s appropriate for the pediatric nurse to cry because of the child/parent’s bad situation.*		
I believe that the tears of a pediatric nurse during a child/parent contact are experienced as very stressful by the pediatric nurse.*		
Crying by a pediatric nurse in the presence of a child/parent might increase the risk of burn out.**		
A pediatric nurse may also cry during a child/parent contact	** − 0.82**	
Tears of a pediatric nurse may be visible to the child/parent	** − 0.80**	
It is disturbing when a pediatric nurse cries during a child/parent contact	**0.79**	
In contact with a child/parent, a pediatric nurse should be as authentic as possible and be him/herself, even if this means that (s)/he sometimes has to cry	** − 0.76**	
A pediatric nurse should feel free to cry during a child/parent contact	** − 0.74**	
When a pediatric nurse cry (s)/he cannot control his/her emotions	**0.70**	
I think it’s appropriate for a pediatric nurse to cry in the presence of a child/parent because of the bad situation of the child/parent	** − 0.70**	
A pediatric nurse who cries in the presence of a child/parent is unprofessional	**0.70**	
During a child/parent contact, professional distance by a pediatric nurse is more important than empathy	**0.68**	
A pediatric nurse who cries during child/parent contact is not suitable as pediatric nurse	**0.67**	
A pediatric nurse who cries in the presence of a child/parent can be a good empathic reaction	** − 0.66**	
I feel uncomfortable when a pediatric nurse cries during a child/parent contact	**0.66**	
When a pediatric nurse cries in the workplace, (s)he cannot handle the job	**0.62**	
I think it’s appropriate for a pediatric nurse to cry in the presence of a child/parent	** − 0.61**	
A pediatric nurse who cries in the presence of a child/parent makes him/herself ridiculous	**0.61**	
A pediatric nurse who cries in the presence of a child/parent has greater risk to make mistakes and/or wrong decisions	**0.58**	
When a pediatric nurse starts crying, I try to get him/her to stop immediately	**0.55**	
A good pediatric nurse should always take distance from the child/parent and his/her problems	**0.53**	
When a pediatric nurse cries during a child/parent contact, (s)he shows inappropriate involvement	**0.50**	
I ignore the crying of a pediatric nurse during a child/parent contact	**0.49**	
Tears of a pediatric nurse in the presence of a child/parent could be important for the contact between child/parent and pediatric nurse	** − 0.49**	
I think it’s appropriate for a pediatric nurse to cry in the presence of a child/parent because of negative performance feedback		**0.89**
I think it’s appropriate for a pediatric nurse to cry in the presence of a child/parent because of a conflict in the work setting		**0.87**
I think it’s appropriate for a pediatric nurse to cry in the presence of a child/parent because of work overload		**0.79**
I think it’s appropriate for a pediatric nurse to cry in the presence of a child/parent because of private circumstances		**0.72**
Eigenvalues	9.09	2.95
% of variance	36.00	12.00
α	0.93	0.83

*Items deleted based on KMO < 0.5. **Items deleted based on factor loading < 0.4

#### Construct validity

A strong correlation was found between the additional question for construct validity purposes and the total score; i.e., Spearman’s rho = 0.75 for pediatricians, for pediatric nurses Spearman’s rho = 0.71.

#### Completeness of the questionnaire

The mean score on the question on completeness of the questionnaire was 7.7 (min–max 1–10, SD = 1.51).

## Discussion

This study describes the development and the reliability—and validity testing of the PACPN questionnaire which measures parents’ attitudes towards crying pediatricians and pediatric nurses. Four items were deleted, resulting in a 25-item questionnaire. Two dimensions could be distinguished, i.e., family circumstances and personal circumstances of the pediatrician or pediatric nurse. The questionnaire showed high internal consistency for the two dimensions. Regarding construct validity, the total score on the questionnaire and having a positive attitude towards crying pediatricians or pediatric nurses were strongly correlated. The mean score of the perceived completeness was 7.7.

The fit of the model was 0.96 for both pediatricians and pediatric nurses, indicating a two-dimension solution seemed appropriate. The two dimensions that were distinguished, relate to either the “family circumstances”, e.g., “I think it’s appropriate for a pediatrician/pediatric nurse to cry in the presence of a child/parent because of the bad situation of the child/parent” or the “personal circumstances” of the HCP, e.g., “I think it’s appropriate for a pediatrician/pediatric nurse to cry in the presence of a child/parent because of work overload.” These findings are underlined by the evaluative study of the original questionnaire [1]. Moreover, this study showed that physicians and medical interns generally felt that it was appropriate to cry at work over the bad circumstances of a patient, but not over their own personal circumstances in the presence of patients [1]. The authors of the original study did not measure the validity. Cronbach alphas of the subparts of the original questionnaire are almost in the same range (0.74–0.90) as our questionnaire (0.81–0.93) [1].

With an average score of 7.7/10.0, there is some room for improvement regarding the completeness of the questionnaire. Several situational factors were associated with physician attitudes on crying in professional settings in front of patients, e.g., delivering bad news or treatment failure [2]. Therefore, adding items about a clearly defined situation might improve the completeness of the questionnaire, e.g., whether the crying occurred during the conversation in which the diagnosis was made. We also expect that including the degree of crying, e.g., moist eyes or crying out loud, will improve the completeness of the questionnaire because this can make a big difference in how crying is perceived [20]. Future follow-up qualitative research should focus on which items are most important for parents in this setting.

Prior research showed that HCPs and students expressed a need for more attention towards crying in training [1, 22]. In addition, medical students have various negative attitudes towards their crying and are often concerned about crying in the presence of patients [22, 23]. This could lead to an unhealthy suppression of their emotions [1]. Although we know that parents of children receiving pediatric palliative care have a clear need for emotional support from HCPs, there is to date only scarce evidence that suggest that the tears of a HCP can be seen as a form of emotional support for parents [11]. Therefore, insights into parents’ attitudes are important to gathering knowledge for the development of an educational module for HCPs. In this education module, we can teach HCPs how to provide emotional support to parents and maintain themselves while providing pediatric palliative care.

### Strength and limitations

This study has several limitations that need to be addressed. Because the online recruitment was based on self-selection, anyone could complete it. This self-selection may have led to biased reliability and validity estimates [24]. Doubts remain about a small group of participants whether they meet the inclusion criteria. For example, seven parents (4%) indicated to have a child for whom no diagnosis has been made. Because in pediatric palliative care, children without a clear diagnosis are relatively common [25], we conducted for validity purposes an analysis with and without a diagnosis, which showed there was no difference in the statistical analysis.

Twenty-seven percent of the parents completed the questionnaire only partially; 30 parents stopped after completing the “experience with crying pediatricians and/or pediatric nurses” questions. It is not clear why they stopped. This could possibly be due to the number of questions (twenty-nine) they had to answer for both pediatricians and pediatric nurses and the associated time needed to complete the questionnaire. However, according to the literature 5–10 min is an acceptable time to fulfill a self-administered questionnaire [26]. Despite these partial responders, our response rate is higher than the commonly used threshold of 60% [27]. Yet, nonresponse bias cannot be completely ruled out, so our results should be interpreted with some caution.

Looking at the demographic background data, there is a gender imbalance in the sample. In this study, mothers were over-represented in the sample (92%) which is a well-known phenomenon in pediatric palliative research [28–35]. In a systematic review based on 45 articles that addressed this gender imbalance, it was shown that 75% of research samples of parents were mothers [36]. However, how a person evaluates crying, appeared to be highly associated to someone’s beliefs about the helpfulness of crying, irrespective of gender [37]. With this in mind, it is interesting to investigate in future research what a more equal distribution of gender does to the reliability and validity of this questionnaire.

The developed PACPN questionnaire was validated for both pediatricians and pediatric nurses. However, the questions regarding these HCPs were not collected separately. The disadvantage of using these so-called juxtaposed scales is that it will elicit different responses than using separate scales [38]. Analysis of the data showed similarities in the responses given for both HCPs, possibly resulting in nearly identical reliability and validity for both pediatricians and pediatric nurses. It is possible that the chosen method of data collection led to results regarding the general attitudes towards crying HCPs instead of the difference in attitudes towards crying pediatricians and pediatric nurses. Finally, another limitation is that we have not measured the cultural backgrounds of the parents. A previous study hypothesized that cultural variation in crying will be predominantly limited to public settings, e.g., hospitals [39]. We hypothesize that the use of separate questionnaires offered at different times, taking into account the variation in cultural background between parents, leads to more heterogeneous responses with different reliability and validity outcomes.

The main strength of this study was the size of the group of parents. Because children who are living with, have died from or survived an LLC or LTC is a small group, it was expected to be challenging to find enough parents. No fewer than 116 parents completed the questionnaire in full. Moreover, the group of included children showed sufficient variance in characteristics like diagnosis and treatment outcome. A third strength was the use of an expert panel to achieve face and content validity.

## Conclusion

The PACPN questionnaire showed good internal consistency and some degree of construct validity regarding parents’ attitudes towards crying pediatricians or pediatric nurses. Future research should reveal further insights into these attitudes. We hypothesize that by adding some items with a clearly defined situation and taking into account the degree of crying will improve the completeness of the questionnaire.

## Data Availability

Anonymized data are available and may be requested from the corresponding author by explaining the rationale.

## Abbreviations

HCPs: Healthcare professionals
KMO: Kaiser-Meyer-Olin
LLC: Life-limiting condition
LTC: Life-threatening-condition
NICU: Neonatal Intensive Care Unit
PACPN: Parents’ Attitudes Towards Crying Pediatricians and pediatric Nurses
PCA: Principal component analysis
PICU: Pediatric intensive care unit
